# Controversy on the treatment of multiple sclerosis and related disorders: positional statement of the expert panel in charge of the 2021 DGN Guideline on diagnosis and treatment of multiple sclerosis, neuromyelitis optica spectrum diseases and MOG-IgG-associated disorders

**DOI:** 10.1186/s42466-021-00139-8

**Published:** 2021-08-06

**Authors:** A. Bayas, A. Berthele, B. Hemmer, C. Warnke, B. Wildemann

**Affiliations:** 1 Universitätsklinikum Augsburg, Klinik für Neurologie und klinische Neurophysiologie, Augsburg, Germany; 2 Technische Universität München, Fakultät für Medizin, Klinikum rechts der Isar, Klinik für Neurologie, Munich, Germany; 3 Klinik und Poliklinik für Neurologie, Medizinische Fakultät, Universitätsklinik Köln, Cologne, Germany; 4 Neurologische Klinik, Universitätsklinikum Heidelberg, Heidelberg, Germany

Guidelines for the diagnosis and treatment of a given disease are intended to ensure high-quality medical care. The German Neurological Society’s (*Deutsche Gesellschaft für Neurologie*, DGN) Guideline on Diagnosis and Treatment of Multiple Sclerosis, Neuromyelitis Optica Spectrum Diseases and MOG-IgG-Associated Disorders [1] has recently been revised and published in German. This consensus-based guideline (S2k level according to the classification of the Association of the Scientific Medical Societies in Germany [*Arbeitsgemeinschaft der Wissenschaftlichen Medizinischen Fachgesellschaften e.V.*, AWMF]) aims to provide answers to questions of daily practice, including areas where evidence is sparse, without limiting physicians’ freedom of decision. A clear focus was set on safeguarding patient autonomy; to this end, patient representatives were involved throughout the entire guideline development process.

## Key question 1. When should patients receive a disease-modifying treatment?

### Before or after the onset of disease progression? Or even after a clinically isolated syndrome (e.g. isolated optic neuritis)?

The selection and timing of disease-modifying drugs (DMDs) in clinically isolated syndromes (CIS) not fulfilling the 2017 diagnostic MS criteria and in multiple sclerosis (MS) is one of the central issues addressed in the DGN guideline [1]. In MS, the prevention of disability accrual is the central goal of all immunomodulatory treatments and there is no doubt that this should start early and according to need.

In the DGN guideline, CIS is defined as a first manifestation (relapse), with the criterion of dissemination in space being fulfilled, but not dissemination in time. The lack of reliable predictive markers for the individual disease course and response to DMDs makes treatment decisions challenging. According to the DGN guideline, the purpose of DMD treatment is to reduce relapse frequency, slow the progression of disability, and decrease disease activity as detected by magnetic resonance imaging (MRI) (see Appendix, statement A14).

In patients who experience a first relapse without fulfilling the criteria of dissemination in time and space (e.g. isolated optic neuritis or myelitis) and therefore cannot be diagnosed with MS or CIS on the basis of the 2017 criteria, DMDs should be used only exceptionally (see A20), since there are no controlled studies on DMDs in this patient group.

A central and strong recommendation in the DGN guideline is to start DMDs in patients diagnosed with CIS or relapsing–remitting MS (RRMS) (see A16), based on the known positive effects of early treatment on the long-term disease course [2]. Permanent disability can and must be avoided by the timely use of DMDs.

This recommendation is in line with the ECTRIMS/EAN guidelines, which recommend that DMDs be offered to patients who have CIS and abnormal findings on MRI, with lesions suggestive of but not fulfilling the criteria for MS, and propose early treatment with DMDs in patients with active RRMS, as defined by clinical relapses and/or MRI activity [3].

Additionally, statement A24 strongly recommends that DMDs be offered to patients with clinical or MRI activity within the previous approximately 2 years.

Beyond the ECTRIMS/EAN guidelines, however, the DGN guideline aims to provide more specific guidance on DMD selection in particular situations, e.g. to offer treatment-naive patients higher-efficacy drugs from the start in the presence of evidence for a *probably highly active* disease course (as outlined in key question 2).

Notably, observational studies [4, 5] have, in some patients, described a mild MS long-term disease course despite the absence of any reliably predictive markers for individual prognosis at disease onset. Taking this into account, the DGN guideline states that one may *consider* (weak recommendation), refraining from DMD initiation, given that the benefits and risks of starting treatment have been thoroughly discussed with the patient, and provided that the patient is monitored closely. The prerequisite for this consideration, according to the guideline, is the assumption of a mild disease course based on patients’ characteristics at the time of disease onset and/or, if available, the subsequent disease course. The relevant characteristics include the severity of and recovery from the first relapse, relapse frequency, MRI lesion load and activity, and cerebrospinal fluid parameters, which should be assessed (see A16). Notwithstanding, the panel feels that several patient characteristics constitute unequivocal reasons for initiation of treatment after the first relapse: young age, polysymptomatic relapse and incomplete recovery from relapse, high lesion load and/or spinal or infratentorial lesions on MRI, and intrathecal synthesis of immunoglobulin G or M.

Given the fact that intrathecal immunoglobulin G or M synthesis is frequent in MS (58.2% for IgG and 21.7% for IgM in a recent study [6] on CIS and MS, based on the 2015 revised McDonald criteria), the option not to initiate a DMD is clearly an exception, applicable only to a small proportion of patients. This number drops further when the other prognostic factors mentioned above are reviewed in individual patients.

Overall, the DGN guideline clearly recommends DMDs for the treatment of patients with MS and CIS. In selected cases, however – in clinical practice, probably few in number – the option not to start DMD treatment first may be considered, provided that the individual patient’s characteristics are indicative of a very favourable disease course and that they are monitored closely. This option was explicitly supported by the patient representatives involved in the panel.

## Key question 2. When should patients receive which disease-modifying treatment?

### Always drugs with limited efficacy but established long-term safety first? Or higher-efficacy drugs for a fraction of patients from the beginning? Individual choice or rigid escalation of treatment?

With more than a dozen DMDs now available, categorisation of these drugs is necessary to facilitate treatment decisions. The panel felt that dichotomisation into baseline vs escalation DMDs is oversimplified, particularly because drugs in the latter category vary widely in efficacy. Seeking a suitable criterion, the panel decided to use the drug-related reduction in relapse rate, available from the pivotal clinical trials. Although the panel fully realises that, owing to differences in baseline characteristics and study design, this measure cannot be compared across studies with full scientific diligence, it represents, in the absence of head-to-head clinical trials for most DMDs, an acceptable approximation. Following this concept, the panel agreed to classify the available DMDs for relapsing MS (RMS) - based on the relative reduction of relapse rate, MRI activity and relapse-related progression (see A17) - into three categories of efficacy (see A18):

- Efficacy category 1: beta-interferons, dimethyl fumarate, glatirameroids, teriflunomide
- Efficacy category 2: cladribine, fingolimod, ozanimod
- Efficacy category 3: alemtuzumab, anti-CD20 antibodies (ocrelizumab, rituximab), natalizumab

**Fig. 1:**
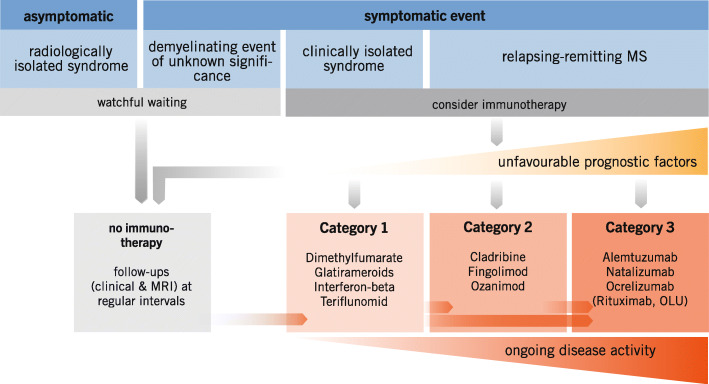
Consensus was reached that category 1 drugs should be used as first-line treatment in DMD-naive patients unless the disease course is considered *probably highly active* (see A25). Efficacy category 2 and 3 DMDs should be used if category 1 drugs have failed to control the disease. However, apart from this well-known escalation approach, category 2 and 3 agents should also be offered to DMD-*naive* patients whose disease course is considered *probably highly active* from the beginning (see A29). The choice of category 1 to 3 thus follows both a step-in and a step-up approach. (OLU, off-label use)

These categories are intended to provide orientation when choosing the appropriate drug for a given disease activity; they do not dictate a “therapeutic ladder”. Instead, immunotherapy for RMS should be selected individually based on the activity of the disease, considering relapse frequency, relapse severity, response to relapse therapy, disease progression and MRI findings (see A23 and Fig. 1).

But what is “probably highly active”? Unfortunately, to date, there is no unequivocal definition of what constitutes a highly active disease, at least not one derived from controlled prospective clinical data. Striving to fill this gap, the panel members suggested criteria based on the findings of the 2018 ECTRIMS Focused Workshop Group on aggressive MS [7] (see A28):

DMD-naive RMS patients meeting one or more of the following clinical criteria are considered as suffering from *probably highly active* MS:

- A relapse has led to clinical deficit affecting activities of daily life despite relapse therapy
- Recovery is insufficient following the first two relapses
- The relapse rate is high, i.e. ≥ 3 in the (approx.) first 2 years or ≥ 2 in the (approx.) first year since disease onset
- EDSS ≥3 in the (approx.) first year since disease onset
- Pyramidal signs in the (approx.) first year since disease onset

The panel agreed that MRI criteria only, without a clinical criterion, are insufficient to assume a *probably highly active* disease course in DMD-naive patients. In the presence of such a criterion, however, the MRI findings (high T2 lesion load, more than one contrast-enhancing lesion, infratentorial/spinal lesions) were considered of high relevance for selecting the first DMD in treatment-naive patients.

In addition, MRI criteria were included in the working definition of an *active* disease despite DMD treatment. In patients with RMS on DMD for more than 6 months the disease is defined as *active* when one or more of the following conditions is fulfilled within the preceding 2 years (see A32):

- Confirmation of a relapse (e.g. by objective clinical findings)
- A relapse and one or more new MS-specific MRI lesion
- At two or more time points, one or more new MS-specific MRI lesions.

This definition is again linked to the strong recommendation (see A33) that patients who have an *active* disease course on treatment with DMD of efficacy category 1 be switched to DMD of efficacy category 2 *or* even 3, depending on the extent of the inflammatory activity.

Thus, the DGN guideline advocates an individual choice of DMD to treat each patient as needed. This may include timely DMD escalation, but DMD titration is by no means mandatory. But why not treat “hard and early”? The panel felt that the results of ongoing prospective studies such as DELIVER-MS [8] or TREAT-MS [9] (not expected until 2023) should be awaited before considering general recommendation of unselected early aggressive treatment.

Instead, the DGN guideline aims at a “treat to target” approach that requires the definition of DMD categories based on efficacy, the definition of *probably highly active* MS in DMD naive patients, and the definition of inflammatory *active* MS in patients on DMD for more than 6 months based on close monitoring throughout the disease course. As such, the guideline assists decisions on which of the growing number of different DMDs can be offered to the individual patient, including clear guidance on when and how to use higher-efficacy drugs from the outset in DMD naive individuals considered to suffer from *probably highly active* MS.

## Key question 3. When should disease-modifying treatment be stopped?

### Generally after a defined time (e.g. 5 years), or later and sometimes not at all? What about reactivation/rebound of disease activation?

Discontinuation of DMD treatment is an important issue that is often addressed in daily practice, in particular by RMS patients who have remained stable for many years both clinically and radiologically while exposed to long-term immunotherapy. Whether it is possible to safely stop treatment in such patients is currently an unresolved question – as it is for patients with inactive secondary progressive MS (SPMS).

Hopefully, three ongoing prospective randomised trials, two including patients with RRMS (DISCOMS [10], estimated completion date February 2022; DOT-MS [11], estimated completion date January 2024) and one including an SPMS population older than 50 years (Stop-I-SEP [12], estimated completion date January 2026), will clarify the impact of treatment discontinuation on focal disease activity and disability progression in the foreseeable future. To date, several observational studies have concluded that discontinuation from (mostly) injectable DMD does not, in general, impact freedom from relapses but may have negative effects on the progression of disability. Age, gender and disability at baseline or acquired within 3 years before withdrawal from treatment (with mostly injectable drugs) determine the risk of not remaining stable in the long term [13–16]. These data are in accordance with circumstantial evidence that the inflammatory component of MS, and consequently the efficacy of DMD, declines with increasing age [17, 18].

Taking into account both the available evidence on withdrawal from treatment and data from observational studies implying a mild long-term disease course [4, 5] in some patients, the DGN guideline panel took the opportunity to draw up recommendations on “treatment duration and discontinuation” (see A60) in highly selected patient groups:

In RMS patients with an anticipated mild disease course at onset (based on clinical and radiological features, i.e. severity of and recovery from first relapse, relapse frequency, MRI lesion load and activity, and CSF parameters [see A16]), and stable disease during the preceding 5 years on category 1 DMD treatment (no further clinical or radiological MS activity or progression), the guideline (a) gives a weak recommendation to pause category 1 DMD treatment; (b) strongly emphasises that patients should be informed that the advocated period of 5 years is not evidence-based and that no data from controlled trials assessing the impact of treatment discontinuation on future relapses and disability progression are available; (c) highlights that the decision on whether to pause or not to pause DMD treatment has to be taken on a strictly individual basis and in consideration of the individual patient’s wishes; and (d) demands regular clinical and MRI assessments to monitor upcoming disease activity (at 6 and 12 months and subsequently every 12 months following discontinuation of treatment (see A65)).

This recommendation only seemingly contradicts the ECTRIMS/EAN guidelines which recommend “to consider continuing a DMD if a patient is stable (clinically and on MRI) and shows no safety or tolerability issues” [3]. Rather, the ECTRIMS/EAN guidelines pass over the question of whether there is ever an alternative to permanent DMD treatment. In contrast, the DGN guideline cautiously broaches the option of DMD discontinuation in a distinct patient population fulfilling the criteria described above.

Interestingly, the DGN guideline revives a former suggestion: The 2006 recommendations on “Escalating Immunomodulatory Therapy of Multiple Sclerosis”, authored by the MSTKG panel, included a similarly weak recommendation that “treatment discontinuation might be considered after at least 3 years of stable disease (no relapses, no disability progression, stable MRI) by taking into consideration the patient’s wish and the prerequisite of patient education and close monitoring” [19].

Regarding category 2 and 3 DMDs, breakthrough or rebound MS activity after cessation of natalizumab, S1P modulators and - problably - CD20 antibodies is, of course, an issue. Thus, the DGN guideline states that complete cessation of these drugs (without substitution) can by no means be recommended, even if patients have been free of any disease activity for 5 years (see A63). More data are needed to fill the knowledge gap on how to proceed in this clinical situation.

## Key question 4. How strongly do regulatory aspects have to be reflected in a recommendation?

### Is it appropriate to have equivalent recommendations for rituximab (off-label) and ocrelizumab and for approved B-cell-depleting therapies (on-label)?

When assessing the efficacy, safety and tolerability of therapeutic agents or measures for a specific disease or treatment situation, medical guidelines are primarily based on the available evidence in its totality. This evidence can come from different sources: in addition to pivotal phase II and III studies, it may also include data from retrospective case series, registries, cohort studies, and “real world” data. In this respect, it is virtually inevitable that recommendations in medical guidelines are not always congruent with the currently approved indications of various drugs. There may even be no alternative to recommending such off-label use (OLU) if no approved drugs are available for a certain medical need. In this respect, the following constellations of possible or obvious off-label uses can be distinguished in guidelines:

- In the absence of alternatives, the guideline recommends OLU of a drug in situations that are serious in terms of the patient’s health.
- The guideline recommends a specific active substance for a particular therapeutic situation, but not all manufacturers’ preparations are explicitly approved for the indication.
- The guideline discusses therapeutic procedures or medications that are in frequent use although there is scant evidence in favour of doing so. In the absence of explicit advice against this practice, the guideline may be interpreted as sanctioning it.
- The guideline recommends a specific therapeutic principle and makes no distinction regarding the approval status of substances that show no difference in key aspects of the mechanism of action.

The lawfulness and feasibility of OLU differ substantially across the EU. German physicians are allowed to initiate OLU, because medical practitioners, as a so-called liberal profession, have the right of free choice of therapy. However, very strict rules apply to OLU in respect of liability issues, patient education and informed consent. In addition, the costs are not per se covered by health insurance - but the insurance funds are by all means empowered to grant reimbursement on a case-to-case basis and upon request.

The DGN guideline recommends OLU in several circumstances:

First, the guideline recommends OLU of drugs if no labelled medications are available. This is often necessary for the treatment of symptoms, e.g. tremor, ataxia, nystagmus or bladder dysfunction, and even standard when it comes to the first-line immunotherapy of neuromyelitis optica spectrum diseases and MOG-antibody-associated diseases.

Second, the guideline advocates the use of drugs that, in the form of branded products from different manufacturers, may be on-label or off-label for a given indication (e.g. gabapentin for spasticity). Similarly, the guideline mentions drugs that have at least partially proven efficacy, but for which the German Federal Joint Committee (G-BA) has decided that they are excluded from reimbursement by the statutory health insurance funds (e.g. phosphodiesterase-5 inhibitors for erectile dysfunction).

Third, the guideline panel decided to group the anti-CD20 antibodies ocrelizumab and rituximab into a substance class, even though rituximab is not approved for the treatment of MS. The main reason for this decision was that the development and clinical evaluation of ocrelizumab are clearly based on the early findings of the phase II studies with rituximab. The two therapeutic antibodies are also nearly identical in terms of their key pharmacological properties, and large cohort studies have demonstrated the long-term efficacy of rituximab in MS treatment [20–22]. Moreover, until ocrelizumab was approved and launched, in specialised centres it was common practice to treat MS patients with rituximab. The guideline group deemed it essential to ensure that the treatment of these patients, as long as they are stable, remains in line with prevailing guidelines – above all because any change in treatment might expose the patient to unjustified risk. Of course, when using rituximab off-label, liability issues and the special reimbursement conditions typical of OLU must be considered. However, as such, initiation and continuation of treatment with rituximab is not medical malpractice [23] and constitutes an option in MS.

## Data Availability

n.a. (there is no primary research data included).

## Appendix

### DGN guideline recommendations referred to in the manuscript

**Recommendation A14** (consensus; agreement of > 75–95% of the panellists): The goals of immunotherapy should be to prevent or reduce clinical disease activity (relapses and disease progression) and to maintain quality of life. A further goal should be the reduction of subclinical disease activity measurable by magnetic resonance imaging. Realistic treatment goals should be agreed upon with the patient before starting treatment.

**Recommendation A16** (consensus): Immunotherapy should be started in patients with CIS or MS. If a mild course can be assumed from the initial presentation and/or the time course, delaying immunotherapy can be considered under close monitoring of the course and after discussion with the patient. The severity of the first episode, the recovery from it, the number of relapses during the course, MRI parameters (lesion load, activity) and CSF parameters should be included in the decision.

**Statement A17** (consensus): based on their relative reduction in inflammatory activity (relapse rate, MRI activity, relapse-related progression), immunotherapeutics can be differentiated into three efficacy categories.

**Recommendation A18** (strong consensus; agreement of > 95% of the panellists): According to their effects on reduction of relapse rate, immunotherapeutics should be classified into three categories:

- Efficacy category 1 (relative reduction in relapse rate compared with placebo of 30–50%): beta-interferons including peg-interferon, dimethyl fumarate (combined analysis of pivotal trials), glatirameroids, teriflunomide
- Efficacy category 2 (relative reduction in relapse rate compared with placebo of 50–60%): cladribine, fingolimod, ozanimod
- Efficacy category 3 (relapse rate reduction of > 60% compared with placebo or > 40% compared with category 1 agents: alemtuzumab, CD20 antibodies (ocrelizumab, rituximab), natalizumab.

**Recommendation A20** (consensus): In the case of a first relapse event in which the DIS and DIT criteria are not met, and therefore the diagnosis of CIS or RRMS cannot be made (e.g. isolated optic neuritis, isolated myelitis), immunotherapy should be given only in exceptional cases.

**Recommendation A23** (consensus): Immunotherapy of RRMS should be based on disease activity (taking into account relapse frequency, relapse severity, response to relapse therapy, disease progression and MRI findings).

**Recommendation A24** (consensus): Untreated patients with RRMS should be offered immunotherapy if

- at least one clinically objectifiable relapse or
- MRI activity

was detectable in the previous 2 years.

**Recommendation A25** (consensus): Because of the lower long-term safety risks, particularly for beta-interferons and glatirameroids, efficacy category 1 agents should generally be used at baseline unless the disease course is considered to be *probably highly active*.

**Statement A28** (consensus): In treatment-naive patients, MS should be considered *probably highly active* if one or more of the following clinical characteristics are present:

- A relapse has resulted in a severe deficit relevant to daily living after the completion of relapse therapy
- Recovery from the first two relapses is poor
- Relapse frequency is high: ≥ 3 in the first 2 years or ≥ 2 in the 1st year after disease onset
- EDSS ≥3.0 in the first year
- Pyramidal tract involvement in the 1st year of the disease

**Recommendation A29** (consensus): Initiation of immunotherapy with agents of efficacy category 2 (fingolimod, cladribine, ozanimod) or 3 (natalizumab, CD20 antibody) should be offered to treatment-naive patients if a *probably highly active* course is present.

**Statement A32** (strong consensus): The course of RMS should be classified as *active* inflammatory if, in treated patients, at any time later than 6 months following the initiation of immunotherapy

- at least one clinically objectifiable relapse, or
- one clinical relapse and one or more new MS-type lesions on MRI, or
- on two or more occasions, one or more new MS-type lesions on MRI

have occurred in a period of up to 2 years.

**Recommendation A33** (consensus): Patients who have an *active* inflammatory course while receiving treatment with substances in efficacy category 1 should be switched to a substance in efficacy category 2 or 3, depending on the extent of disease activity. As with substances in efficacy category 1, patient-specific aspects (including side effects, type of application, monitoring, duration of action, comorbidities) should be taken into account when choosing a drug from efficacy categories 2 and 3. A switch within category 1 agents or to higher-dose beta interferons should only be considered if patient-specific factors argue against a switch to category 2/3.

**Recommendation A60** (strong consensus): In patients who had low disease activity before initiation of immunotherapy and have not shown disease activity under previous therapy with an efficacy category 1 drug, cessation of treatment may be considered after a period of at least 5 years at the patient’s request. Patients should be informed that the period of 5 years is not based on evidence and there have been no controlled discontinuation studies to reliably assess the risk of re-exacerbation of disease after discontinuation.

**Statement A63** (strong consensus): Currently, no general recommendation can be made to “de-escalate” treatment with natalizumab (in continued JCV-AK-negative patients), S1P modulators, or CD20 antibodies, even if patients on treatment have shown no disease activity for, say, 5 years.

**Recommendation A65** (consensus): If the patient and physician decide to “de-escalate” or pause treatment after weighing all risks, clinical and MRI follow-up should be performed six and 12 months later and at 12-month intervals thereafter. If disease activity is detected (see definition of active inflammatory RMS), resumption or re-escalation of immunotherapy should occur.
